# Supplementary material for: Histone tails cooperate to control the breathing of genomic nucleosomes
Source: PLoS Comput Biol. 2021 Jun 3;17(6):e1009013. doi: 10.1371/journal.pcbi.1009013 (PMC8174689; doi:10.1371/journal.pcbi.1009013)
Supplement: S1 Methods — (PDF) [file pcbi.1009013.s001.pdf]

S1 Methods

Principal Component Analysis

Principal Component Analysis (PCA) was performed using cpptraj. Due to the high flexibility of the histone tails, all histone tails except for the H3 and H2AC tails were removed for the PCA calculation. Non-hydrogen atoms of the DNA and histones (excluding the H4, H2B and H2AN tails) were used. For both the $Esrrb^{hH}$ and the $Lin28^{dH}$ nucleosomes, the covariance matrix was calculated and diagonalized to extract the first 25 eigenvectors and eigenvalues. The trajectories were projected on the first mode, and the minimum and maximum projection values for that mode were extracted. Finally, pseudotrajectories along the first mode were generated to analyze the correlated motions of the L-DNA and tails. PC1 represents 42.93% and 31.3% of all the motions in the $Esrrb^{hH}$ and $Lin28^{dH}$ respectively.

MD simulations of closed nucleosomes with selected H3 and H2AC tail configurations

To reproduce the nucleosome opening of the $Esrrb^{hH}$ and the $Lin28^{dH}$ nucleosomes we selected closed nucleosome conformations from the representative structures in the corresponding clusters. In these we exchanged the H3 and H2AC monomers at the opening end with monomers from representative nucleosome structures of the clusters with open conformations. After mapping the selection criteria on the simulations, for $Esrrb^{hH}$ we selected the nucleosome after 665.5 ns of simulation 1 and the H3 and H2AC monomers after 801 ns of the simulation 1. For $Lin28^{dH}$ we selected the nucleosome after the equilibration of simulation 3 and the H3 and H2AC monomers after 705.3 ns of simulation 2. We solvated, energy minimized and equilibrated these models as described in the main text. Then, we performed 3 independent. unbiased classical MD simulations, each 500 ns long with the protocol described in the main text.

Because $Esrrb^{hH}$ remained closed in these simulations due to interactions between part of the H3 tail and the outer DNA gyre which reformed rapidly during the equilibration, we took an alternative approach. We applied harmonic restraints in an additional 8 ns equilibration on four order parameters (collective variables). These were the minimial interatomic distances ($\delta_{\min}$) between the H3 and H2AC tails and the outer DNA gyre as described in the main text (see Methods) and the corresponding coordination numbers that reflect the number of contacts between the tails and the outer gyre. The coordination number between two groups of atoms, as defined in the Collective Variables Module available in NAMD (https://colvars.github.io/) is:

$$C(group1, group2) = \sum_{i\epsilon group1} \sum_{i\epsilon group2} \frac{1 - (|x_i - x_j|/d_0)^n}{1 - (|x_i - x_j|/d_0)^m}$$

where x_i, x_j are the atomic coordinates of atoms i and j, d_0 is the threshold distance (4.0 Å), n and m are exponents that control the properties of the function for which we used the default values n=6, m=12.

$\delta_{\min}$ and C were changed using a steered MD bias from the initial values to the target values of 13 and 0.5 during 8 ns equilibration which was performed in NAMD using the Collective Variables Module. After this biased equilibration, the structure remained in closed conformation (R_g=48) but no interactions between the H3 and H2AC tails and the outer DNA gyre were present.

Then, we performed three unrestrained classical MD simulations starting with the nucleosome structure at the end of the biased equilibration. First 30 ns of each simulation were considered as further equilibration and were excluded from the analysis.
