# Supplementary material for: Histone tails cooperate to control the breathing of genomic nucleosomes
Source: PLoS Comput Biol. 2021 Jun 3;17(6):e1009013. doi: 10.1371/journal.pcbi.1009013 (PMC8174689; doi:10.1371/journal.pcbi.1009013)
Supplement: S1 Text — (PDF) [file pcbi.1009013.s002.pdf]

S1 Text

Overview of supporting information and supporting data

S1 Document: Alignment of Drosophila and human histones.

S2 Document: DNA sequences.

S1 Data:
Data file in Excel format (bzip2 compressed) with the time series (every 40 ps) of R_g and γ angles. The document has 2 sheets titled by the properties they contain. Each column contains the data from one simulation ensemble with the individual simulations following each other in rows. Each row represents a simulation frame.

S2 Data:
Data file in Excel format (bzip2 compressed) with the time series (every 100 ps) of the median interatomic distances and the number of contacts between the H3 and H2AC tails and different DNA segments. The document has 4 sheets titled by the properties and simulations (dH or hH) they contain. Each column contains the data from one simulation ensemble with the individual simulations following each other in rows. Each row represents a simulation frame.

S1 Fig: time series of the histone-DNA contacts supporting data in Table 1.

S2 Fig: time series and histograms of the γ angles supporting the data in Fig 2.

S3 Fig: time series of the R_g of the histone tails supporting the data in Fig 4.

S4 Fig: distributions of the γ angles in the DNA clusters supporting the data in Fig 5.

S5 Fig: supporting results from the MD simulations started with closed nucleosome conformations but with H3 and H2AC monomers from open nucleosomes. These data is in support of the data in Figs 5 and 6.

Supporting Results

From the first set of unbiased simulations we could reproduce the extensive opening of $Lin28^{dH}$ in two out of three simulations (simulations 2 and 3 in S5 Fig). In the third simulation, $Lin28^{dH}$ was open only in the direction defined by γ_2 (simulation 1 in S5 Fig). However, $Esrrb^{hH}$ remained closed because interactions between part of the H3 tail and the outer DNA gyre were reformed rapidly during the equilibration. This was due to the selected conformation of $Esrrb^{hH}$ which was very closed ($R_g = 46$) and due to some interactions between the H3 tail and the outer gyre that were present in the model.

From the set of simulations started after the biased equilibration, we could also reproduce the extensive opening of $Esrrb^{hH}$. In one of the three simulations $Esrrb^{hH}$ opened very rapidly, and more extensively than in the original simulation (simulation 1 in S5 Fig). The opening was reversible, occuring twice in this simulation. In a second simulation $Esrrb^{hH}$ opened reversibly a few times and stabilized to a conformation with R_g larger than 50 which remained stable during the simulation (simulation 3 in S5 Fig). In both simulations, few interactions between the H3 and H2AC tails lead to nucleosome closing, further supporing

our conclusion that these two tails control nucleoosme breathing. In the third simulation, the nucleosome remained closed, again because a larger number of interactions between the H3 and H2AC tails with the outer DNA gyre reformed.

S6 Fig: Principal Component Analysis supporting data in Fig 7.

Supporting Results
This analysis revealed that in the lowest frequency mode (largest amplitude motion), nucleosome breathing is correlated with the motions of the H3 and H2AC tails (S6 Fig). This further supports our findings that ample nucleosome breathing motions are modulated by transitions in the positions and conformations of the H3 and H2AC tails.

S7 Fig: time series of the minimal interatomic distances (δ_{min} between the H3 tail and the outer and inner DNA gyres supporting data in Fig 8

S8 Fig: Time series of the minimal interatomic distances (δ_{min} between the H2AC tail and the outer and inner DNA gyres supporting data in Fig 8
