# Supplementary material for: Histone tails cooperate to control the breathing of genomic nucleosomes
Source: PLoS Comput Biol. 2021 Jun 3;17(6):e1009013. doi: 10.1371/journal.pcbi.1009013 (PMC8174689; doi:10.1371/journal.pcbi.1009013)
Supplement: S1 Document — (PDF) [file pcbi.1009013.s003.pdf]

S1 Document

Histone alignments

H3

```
                                N-terminal tail
Human       MARTKQTARKSTGGKAPRKQLATKAARKSAPATGGVKKPHRYRPGTVALR
Drosophila  MARTKQTARKSTGGKAPRKQLATKAARKSAPATGGVKKPHRYRPGTVALR

Human       EIRRYQKSTELLIRKLPFQRLVREIAQDFKTDLRFQSSAVMALQEACEAY
Drosophila  EIRRYQKSTELLIRKLPFQRLVREIAQDFKTDLRFQSSAVMALQEASEAY

Human       LVGLFEDTNLCAIHAKRVTIMPKDIQLARRIRGERA
Drosophila  LVGLFEDTNLCAIHAKRVTIMPKDIQLARRIRGERA
```

H4

```
                  N-terminal tail
Human       MSGRGKGGKGLGKGGAKRHRKVLRDNIQGITKPAIRRLARRGGVKRISGL
Drosophila  MTGRGKGGKGLGKGGAKRHRKVLRDNIQGITKPAIRRLARRGGVKRISGL

Human       IYEETRGVLKVFLENVIRDAVTYTEHAKRKTVTAMDVVYALKRQGRTLYG
Drosophila  IYEETRGVLKVFLENVIRDAVTYTEHAKRKTVTAMDVVYALKRQGRTLYG

Human       FGG
Drosophila  FGG
```

H2A

```
              N-terminal tail
Human   MSGRGKQGGKARAKAKTRSSRAGLQFPVGRVHRLLRKGNYSERVGAGAPVYLAAV
Droso   MSGRGK.GGKVKGKAKSRSNRAGLQFPVGRIHRLLRKGNYAERVGAGAPVYLAAV

Human   LEYLTAEILELAGNAARDNKKTRIIPRHLQLAIRNDEELNKLLGRVTIAQGGVLP
Droso   MEYLAAEVLELAGNAARDNKKTRIIPRHLQLAIRNDEELNKLLSGVTIAQGGVLP

                    C-terminal tail
Human   NIQAVLLPKKTESHHKAKGK
Droso   NIQAVLLPKKTEKKA.....
```

H2B

N-terminal tail

```
Human   MPEPAKSAPAPKKGSKKAVTKAQKKDGKKRKRSRKESYSVYVYKVLKQVHPDTGI
Droso   MP.PKTSGKAAKKAG.KAQKNITKTD.KKKKRKRKESYAIYIYKVLKQVHPDTGI

Human   SSKAMGIMNSFVNDIFERIAGEASRLAHYNKRSTITSREIQTAVRLLLPGELAKH
Droso   SSKAMSIMNSFVNDIFERIAAEASRLAHYNKRSTITSREIQTAVRLLLPGELAKH

Human   AVSEGTKAVTKYTSSK
Droso   AVSEGTKAVTKYTSSK
```
