# Supplementary material for: Histone tails cooperate to control the breathing of genomic nucleosomes
Source: PLoS Comput Biol. 2021 Jun 3;17(6):e1009013. doi: 10.1371/journal.pcbi.1009013 (PMC8174689; doi:10.1371/journal.pcbi.1009013)
Supplement: S2 Document — (PDF) [file pcbi.1009013.s004.pdf]

S2 Document

DNA sequences of the Widom, Esrrb and Lin28B nucleosomes.

Widom

```
  1 ACGCGGCCGC CCTGGAGAAT CCCGGTGCCG AGGCCGCTCA ATTGGTCGTA GCAAGCTCTA
 61 GCACCGCTTA AACGCACGTA CGCGCTGTCC CCCGCGTTTT AACCGCCAAG GGGATTACTC
121 CCTAGTCTCC AGGCACGTGT CAGATATATA CATCCTGTGC ATGTATTG
```

Esrrb

```
  1 ATCAGCAGGG AGAAGGAGCG CCTCCCCATG TGGGACCTGG AGAAACAGAG GGTGGAGGGA
 61 GCATAGAGAG TCTGTTCTAA GCTGCAAAGC AAAGGCCTGG CGACCTAGGA GACCATGGAG
121 TTCCAGAAAG TGATAGTTAT GCAGAGCGAA TGGAGGGAAT CAGCACGC
```

Lin28b (Drosophila histones simulations)

```
  1 AGTTAAGTGG TATTAACATA TCCTCAGTGG TGAGTATTAA CATGGAACTT ACTCCAACAA
 61 TACAGATGCT GAATAAATGT AGTCTAAGTG AAGGAAGAAG GAAAGGTGGG AGCTGCCATC
121 ACTCAGAATT GTCCAGCAGG GATTGTGCAA GCTTGTGAAT AAAGACAC
```

Lin28b (human histones simulations)

```
  1 AAGTTAAGTG GTATTAACAT ATCCTCAGTG GTGAGTATTA ACATGGAACT TACTCCAACA
 61 ATACAGATGC TGAATAAATG TAGTCTAAGT GAAGGAAGAA GGAAAGGTGG GAGCTGCCAT
121 CACTCAGAAT TGTCCAGCAG GGATTGTGCA AGCTTGTGAA TAAAGACA
```
