# Supplementary material for: Histone tails cooperate to control the breathing of genomic nucleosomes
Source: PLoS Comput Biol. 2021 Jun 3;17(6):e1009013. doi: 10.1371/journal.pcbi.1009013 (PMC8174689; doi:10.1371/journal.pcbi.1009013)

**S1 Figure** : Number of histone-DNA contacts. The 2 panels depict the time evolution of the number of contacts of the histone core (top) and histone tails (middle and bottom) to the DNA. For the histone tails, contacts were split between contacts to the inner gyre (middle) and contacts to the outer gyre (bottom). (A) Contact evolution in the hH simulations. (B) Contact evolution in the dH simulations. A contact was defined as a non-hydrogem atom closer than 4.5 Å to another non-hydrogen atom.

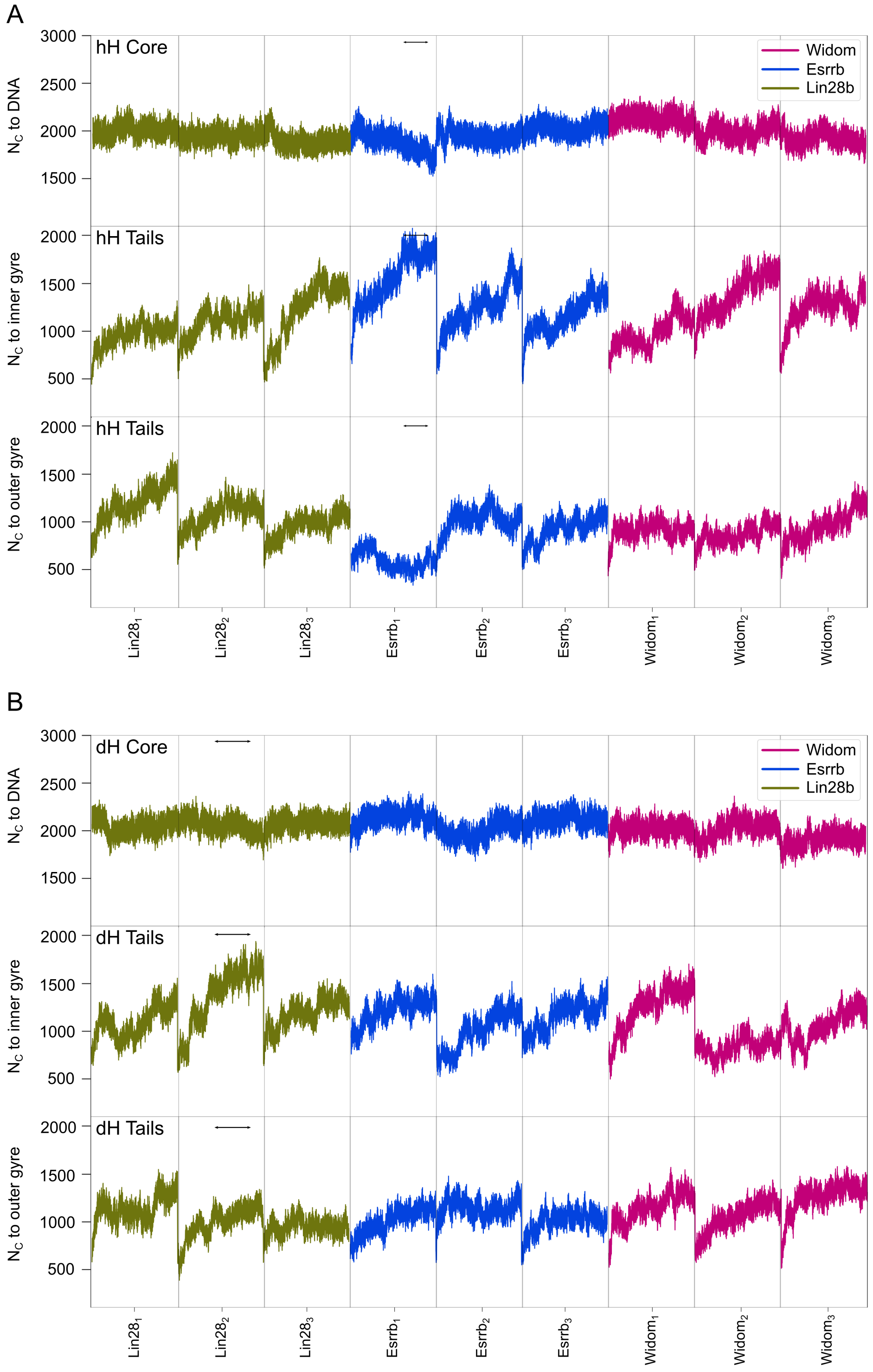

Supplement: S1 Fig — The 2 panels depict the time evolution of the number of contacts of the histone core (top) and histone tails (middle and bottom) to the DNA. For the histone tails, contacts were split between contacts to the inner gyre (middle) and contacts to the outer gyre (bottom). (A) Contact evolution in the hH simulations. (B) Contact evolution in the dH simulations. A contact was defined as a non-hydrogem atom closer than 4.5 Å to another non-hydrogen atom. (PDF) [file pcbi.1009013.s007.pdf]
