# Supplementary material for: Histone tails cooperate to control the breathing of genomic nucleosomes
Source: PLoS Comput Biol. 2021 Jun 3;17(6):e1009013. doi: 10.1371/journal.pcbi.1009013 (PMC8174689; doi:10.1371/journal.pcbi.1009013)

S2 Figure : Nucleosome structural flexibility. (A-B) The evolution of the RoG of the DNA over time, during the hH (A) and dH (B) simulations. (C-F) Time series and histograms of the γ angles described in Figure 2, for the 5' arm of the hH nucleosomes (C), 5' arm of the dH nucleosomes (D), 3' arm of the hH nucleosomes (E) and 3' arm of the dH nucleosomes (F). The individual simulations are separated by vertical black lines.

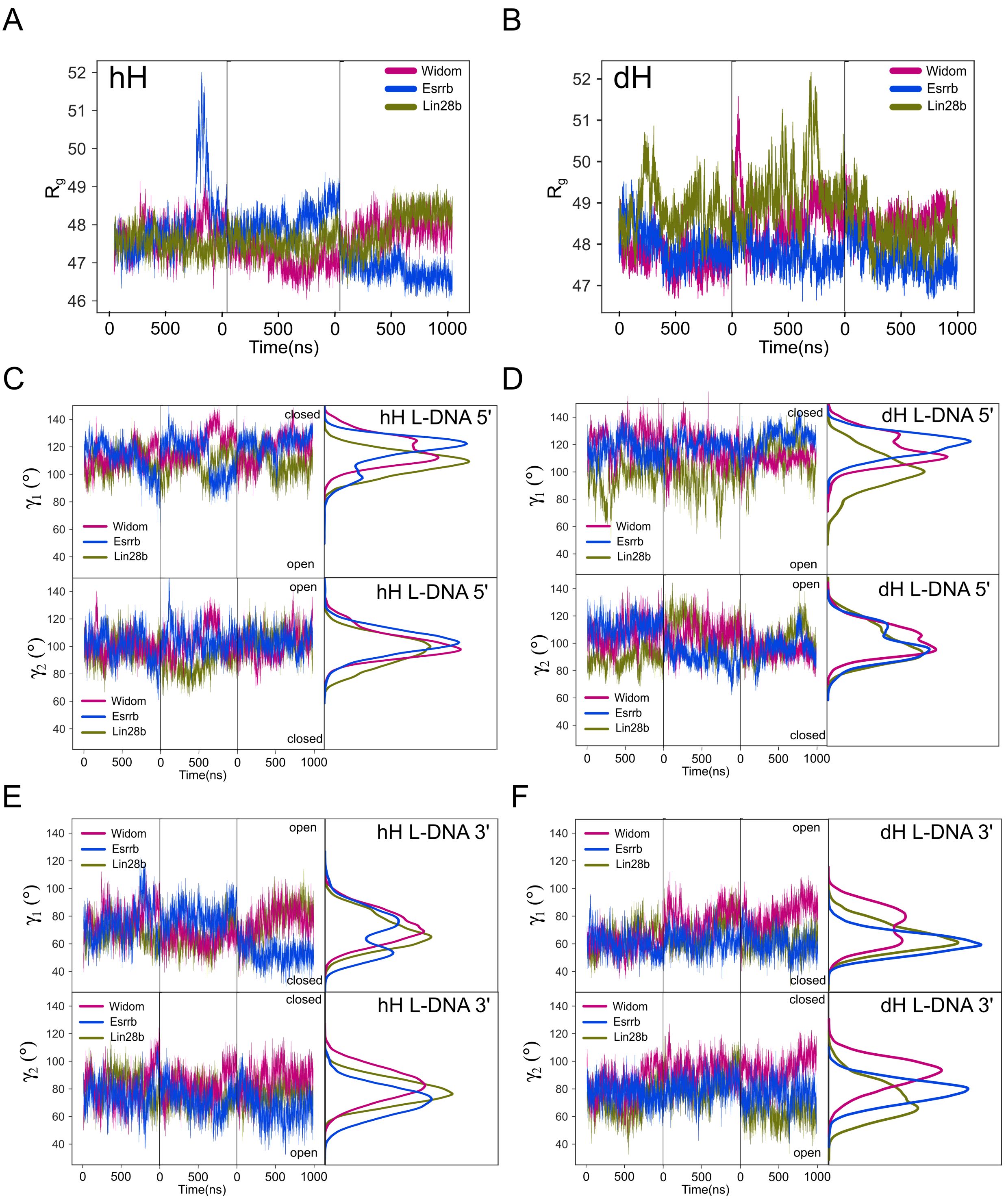

Supplement: S2 Fig — (A-B): The evolution of the Rg of the DNA over time, during the hH (A) and dH (B) simulations. (C-F) Time series and histograms of the γ angles described in Fig 2, for the 5’ arm of the hH nucleosomes (C), 5’ arm of the dH nucleosomes (D), 3’ arm of the hH nucleosomes (E) and 3’ arm of the dH nucleosomes (F). The individual simulations are separated by vertical black lines. (PDF) [file pcbi.1009013.s008.pdf]
