# Supplementary material for: Histone tails cooperate to control the breathing of genomic nucleosomes
Source: PLoS Comput Biol. 2021 Jun 3;17(6):e1009013. doi: 10.1371/journal.pcbi.1009013 (PMC8174689; doi:10.1371/journal.pcbi.1009013)

S3 Figure : Radius of gyration of the histone tails. The evolution of the RoG over time for histones H3 and H2AC tails. (A) hH simulations. (B) dH simulations.

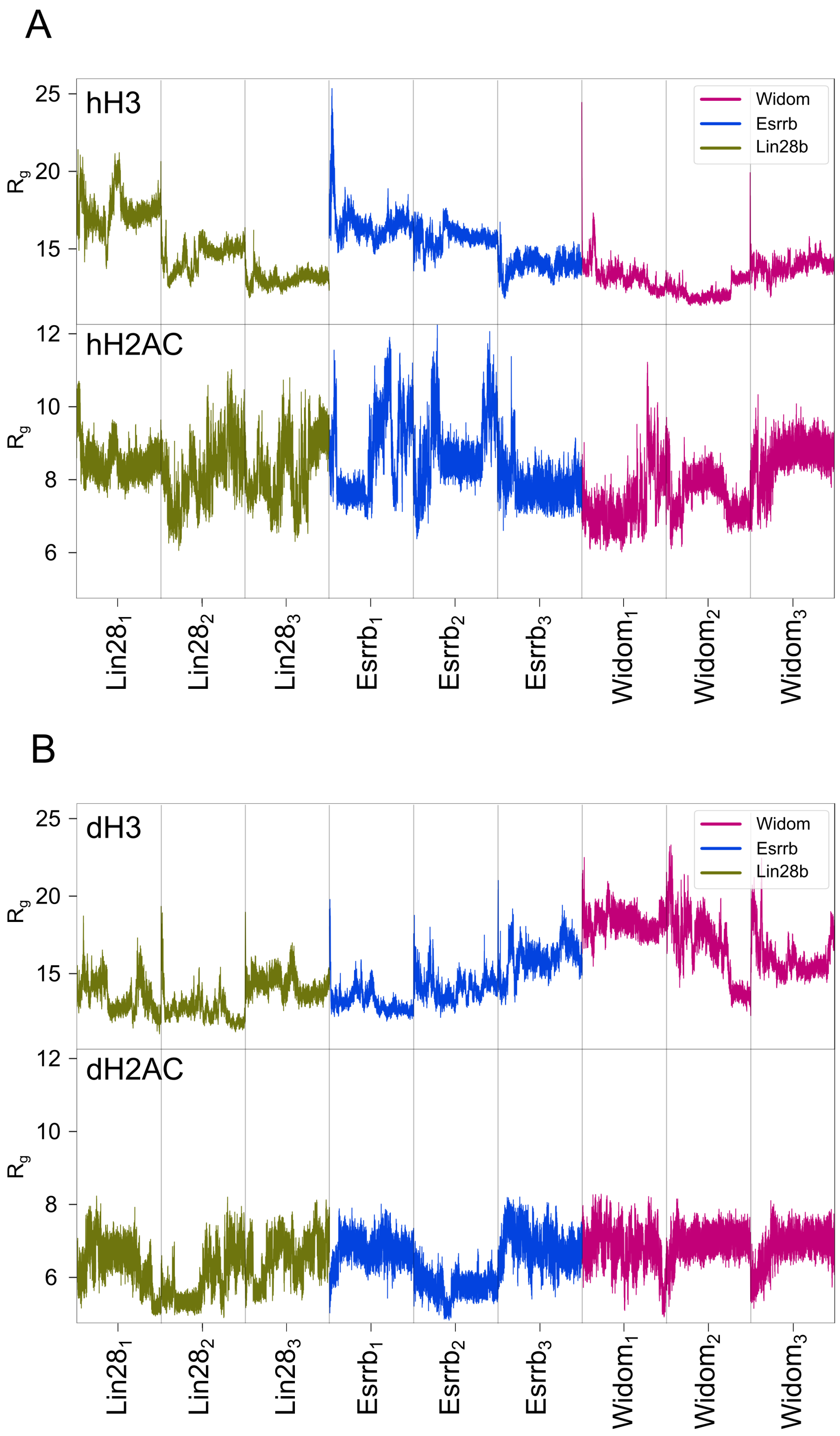

Supplement: S3 Fig — The evolution of the Rg over time for histones H3 and H2AC tails. (A) hH simulations. (B) dH simulations. (PDF) [file pcbi.1009013.s009.pdf]
