# Supplementary material for: Histone tails cooperate to control the breathing of genomic nucleosomes
Source: PLoS Comput Biol. 2021 Jun 3;17(6):e1009013. doi: 10.1371/journal.pcbi.1009013 (PMC8174689; doi:10.1371/journal.pcbi.1009013)

S4 Figure : Sampling of the DNA clusters. Two-dimensional histograms depicting the conformational sampling of the L-DNA arms in the space defined by the γ_1 and γ_2 angles for each individual DNA cluster. (A) 3' L-DNA clusters from the hH simulations. (B) 5' L-DNA clusters from the dH simulations.

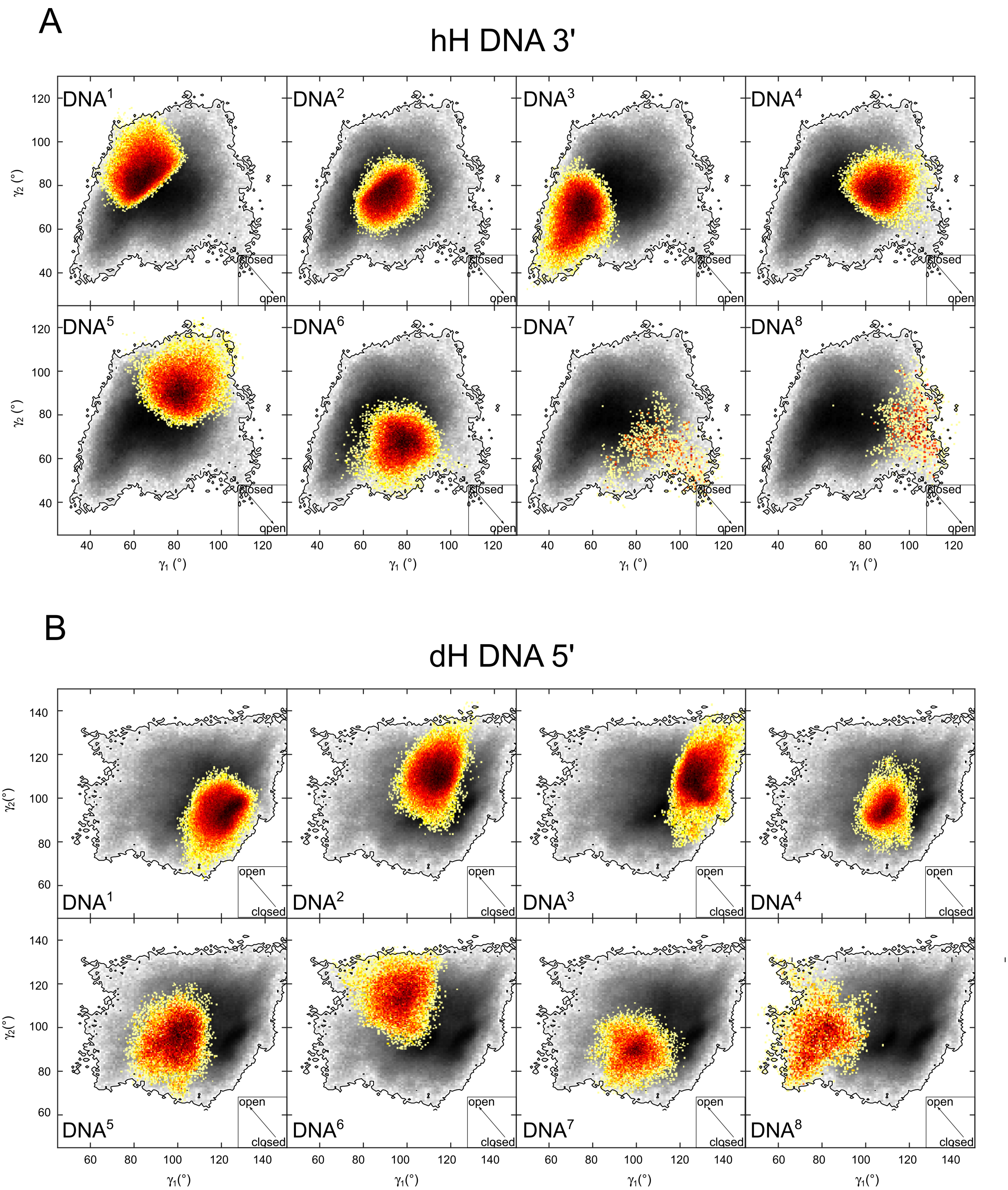

Supplement: S4 Fig — Two-dimensional histograms depicting the conformational sampling of the L-DNA arms in the space defined by the γ1 and γ2 angles for each individual DNA cluster. (A) 3’ L-DNA clusters from the hH simulations. (B) 5’ L-DNA clusters from the dH simulations. (PDF) [file pcbi.1009013.s010.pdf]
