# Supplementary material for: Histone tails cooperate to control the breathing of genomic nucleosomes
Source: PLoS Comput Biol. 2021 Jun 3;17(6):e1009013. doi: 10.1371/journal.pcbi.1009013 (PMC8174689; doi:10.1371/journal.pcbi.1009013)

S5 Figure : Nucleosome opening in simulations with selected nucleosome and histone tail conformations. (A) Table summarizing the simulations we performed to probe the reproducibility of the extensive nucleosome opening (similar to main Table 1). (B-C) Two-dimensional histograms depicting the conformational sampling of the L-DNA arms in the space defined by the γ_1 and γ_2 angles for the Esrrb^{hH} (B) and Lin28b^{dH} (C) nucleosomes. In black are the original histograms (see Figure 2), in green the combined sampling of the three simulations started with a closed nucleosome but with H3 and H2AC tails in configurations found in open nucleosomes. Each simulation is depicted by blue, yellow, and red contours. (D-E) Time series for the R_g during the three 0.5 μs simulations of Esrrb^{hH} (D) and Lin28b^{dH} (E).

A

Histones	DNA	Replica	Time	R_g[1]	Number of contacts[2] with DNA		
					Histone Core	Histone Tails	
						inner gyre[3]	outer gyre[3]
Drosophila	Lin28b	1	500 ns	47.84–49.38	1951–2166	925–1333	930–1262
Drosophila	Lin28b	2	500 ns	48.58–50.03	1907–2154	1070–1607	772–1163
Drosophila	Lin28b	3	500 ns	49.33–50.90	1843–2062	946–1430	880–1230
Human	Esrrb	1	500 ns	49.05–51.61	1819–2008	1147–1620	562–799
Human	Esrrb	2	500 ns	47.77–48.96	1853–2112	1101–1648	561–978
Human	Esrrb	3	500 ns	48.56–50.96	1813–2007	948–1440	675–957

[1] R_g and the number of contacts are shown as the percentiles 5 to 95.
[2] A contact was defined as a non–hydrogen atom closer than 4.5 Å to another non-hydrogen atom.
[3] Inner and outer gyre of the nucleosomal DNA, see Methods for details.

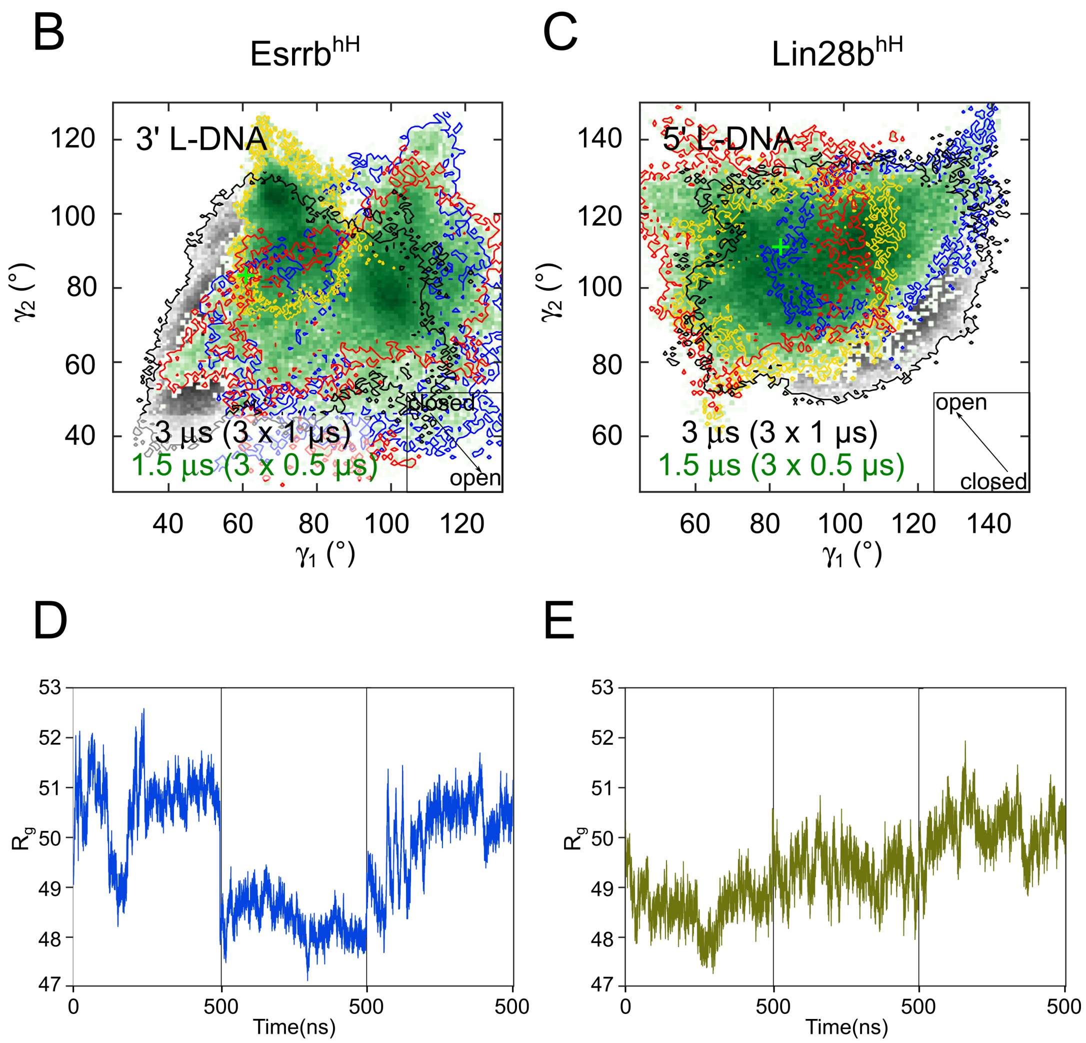

Supplement: S5 Fig — (A) Table summarizing the simulations we performed to probe the reproducibility of the extensive nucleosome opening (similar to main Table 1). (B-C) Two-dimensional histograms depicting the conformational sampling of the L-DNA arms in the space defined by the γ1 and γ2 angles for the EsrrbhH (B) and Lin28bdH (C) nucleosomes. In black are the original histograms (see Fig 2), in green the combined sampling of the three simulations started with a closed nucleosome but with H3 and H2AC tails in configurations found in open nucleosomes. Each simulation is depicted by blue, yellow, and red contours. (D-E) Time series for the Rg during the three 0.5 μs simulations of EsrrbhH (D) and Lin28bdH (E). (PDF) [file pcbi.1009013.s011.pdf]
