# Supplementary material for: Histone tails cooperate to control the breathing of genomic nucleosomes
Source: PLoS Comput Biol. 2021 Jun 3;17(6):e1009013. doi: 10.1371/journal.pcbi.1009013 (PMC8174689; doi:10.1371/journal.pcbi.1009013)

S6 Figure : Correlated motions of the histone tails and linker DNA. (A-D) Superposition of DNA and H3 (A-B) or DNA and H2AC (C-D) snapshots from the pseudotrajectory of the lowest frequency principal component (PC1) of the simulation ensembles of Esrrb^{hH} (A,C) and Lin28b^{dH} (B,D). **E-F)** The RoG along PC1 pseudotrajectory of Esrrb^{hH} (E) and Lin28b^{dH} (F). The closed to open transition is indicated by the Red-White-Blue color scale. The amplitude was normalized such as -1 corresponds to the most closed and +1 to the most open conformation.

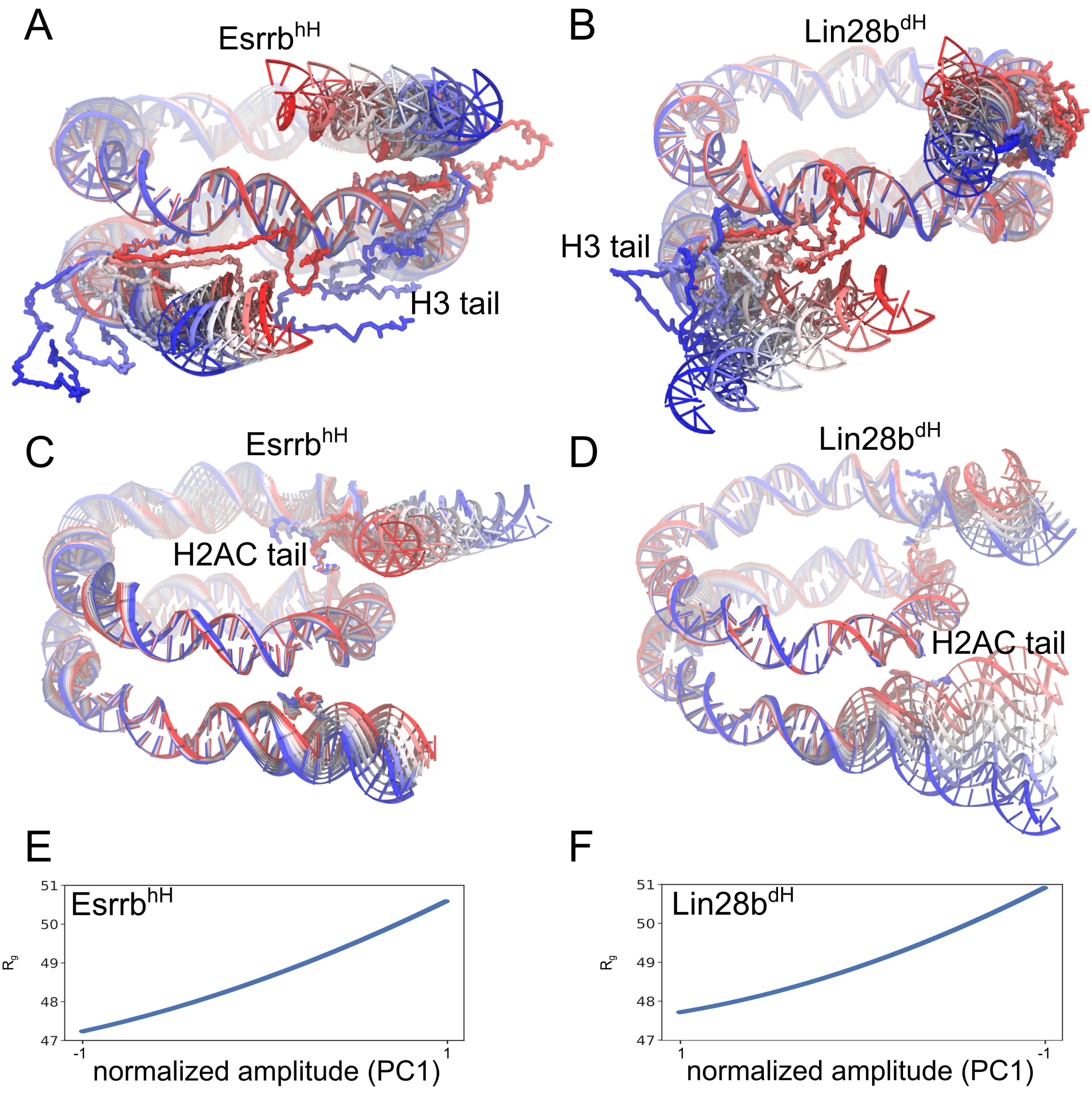

Supplement: S6 Fig — (A-D) Superposition of DNA and H3 (A-B) or DNA and H2AC (C-D) snapshots from the pseudotrajectory of the lowest frequency principal component (PC1) of the simulation ensembles of EsrrbhH (A,C) and Lin28bdH (B,D). (E-F) The Rg along PC1 pseudotrajectory of EsrrbhH (E) and Lin28bdH (F). The closed to open transition is indicated by the Red-White-Blue color scale. The amplitude was normalized such as -1 corresponds to the most closed and +1 to the most open conformation. (PDF) [file pcbi.1009013.s012.pdf]
