# Supplementary material for: Histone tails cooperate to control the breathing of genomic nucleosomes
Source: PLoS Comput Biol. 2021 Jun 3;17(6):e1009013. doi: 10.1371/journal.pcbi.1009013 (PMC8174689; doi:10.1371/journal.pcbi.1009013)

S7 Figure : Interactions of H3 residues with DNA. The evolution of the H3 residues position relative to the inner and outer gyre of the DNA. (A) The Esrrb^{hH} nucleosome. (B) The Lin28b^{dH} nucleosome. The plot at the top row shows the nucleosome RoG to monitor opening and closing events. The other plots show the minimal distance of the residues to the outer gyre, colored by the minimal distance to the inner gyre of the DNA.

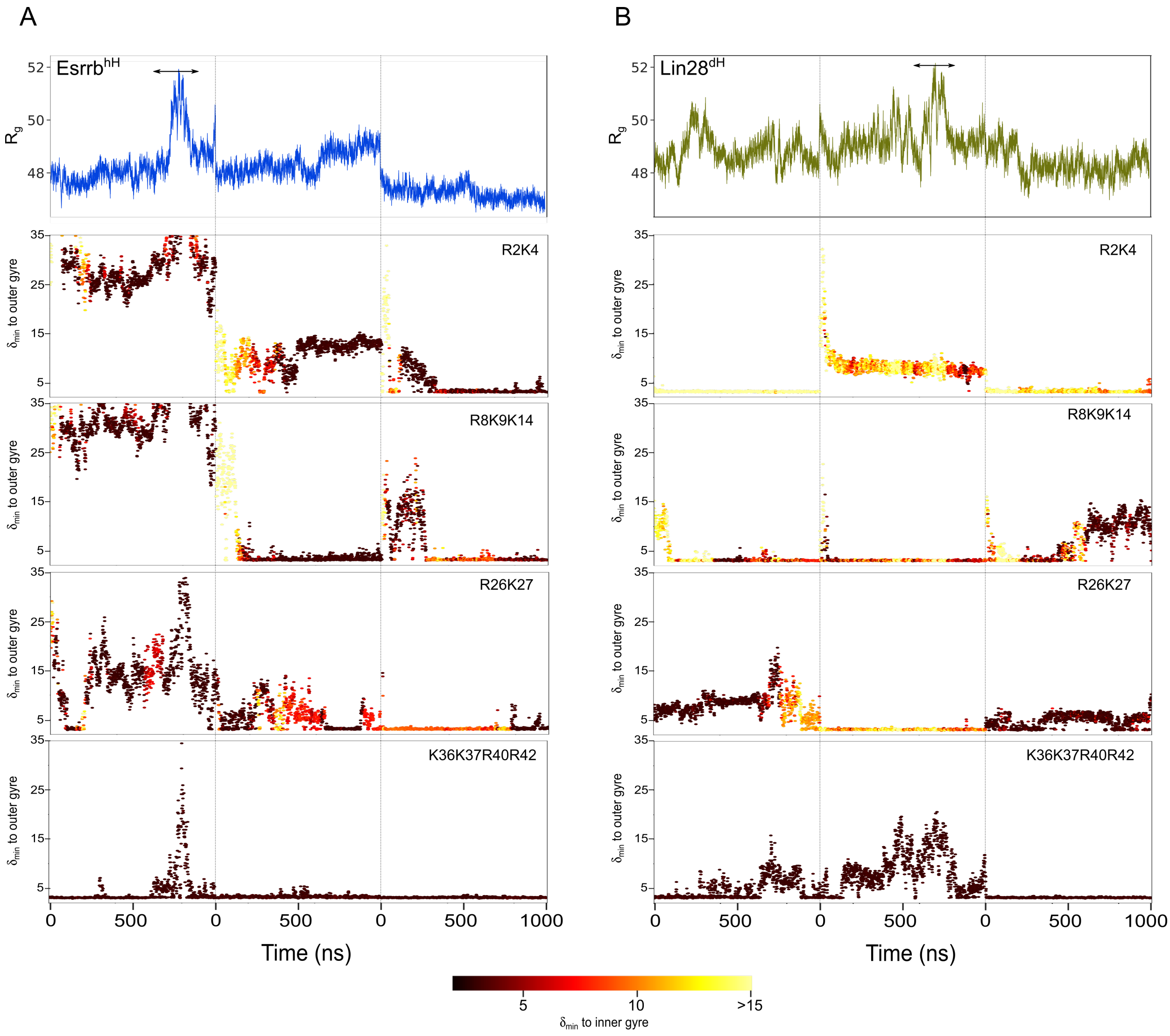

Supplement: S7 Fig — The evolution of the H3 residues position relative to the inner and outer gyre of the DNA. (A) The EsrrbhH nucleosome. (B) The Lin28bdH nucleosome. The plot at the top row shows the nucleosome Rg to monitor opening and closing events. The other plots show the minimal distance of the residues to the outer gyre, colored by the minimal distance to the inner gyre of the DNA. (PDF) [file pcbi.1009013.s013.pdf]
