# Supplementary material for: Histone tails cooperate to control the breathing of genomic nucleosomes
Source: PLoS Comput Biol. 2021 Jun 3;17(6):e1009013. doi: 10.1371/journal.pcbi.1009013 (PMC8174689; doi:10.1371/journal.pcbi.1009013)

S8 Figure : Interactions of H2AC residues with DNA in the nucleosomes with large opening. The evolution of the H2AC residues position relative to the inner and outer gyre. (A) The Esrrb^{hH} nucleosome. (B) The Lin28b^{dH} nucleosome. The plot at the top row shows the nucleosome radius of gyration to monitor opening and closing events. The other plots show the minimal distance of the residues to the outer gyre, colored by the minimal distance to the inner gyre of the DNA.

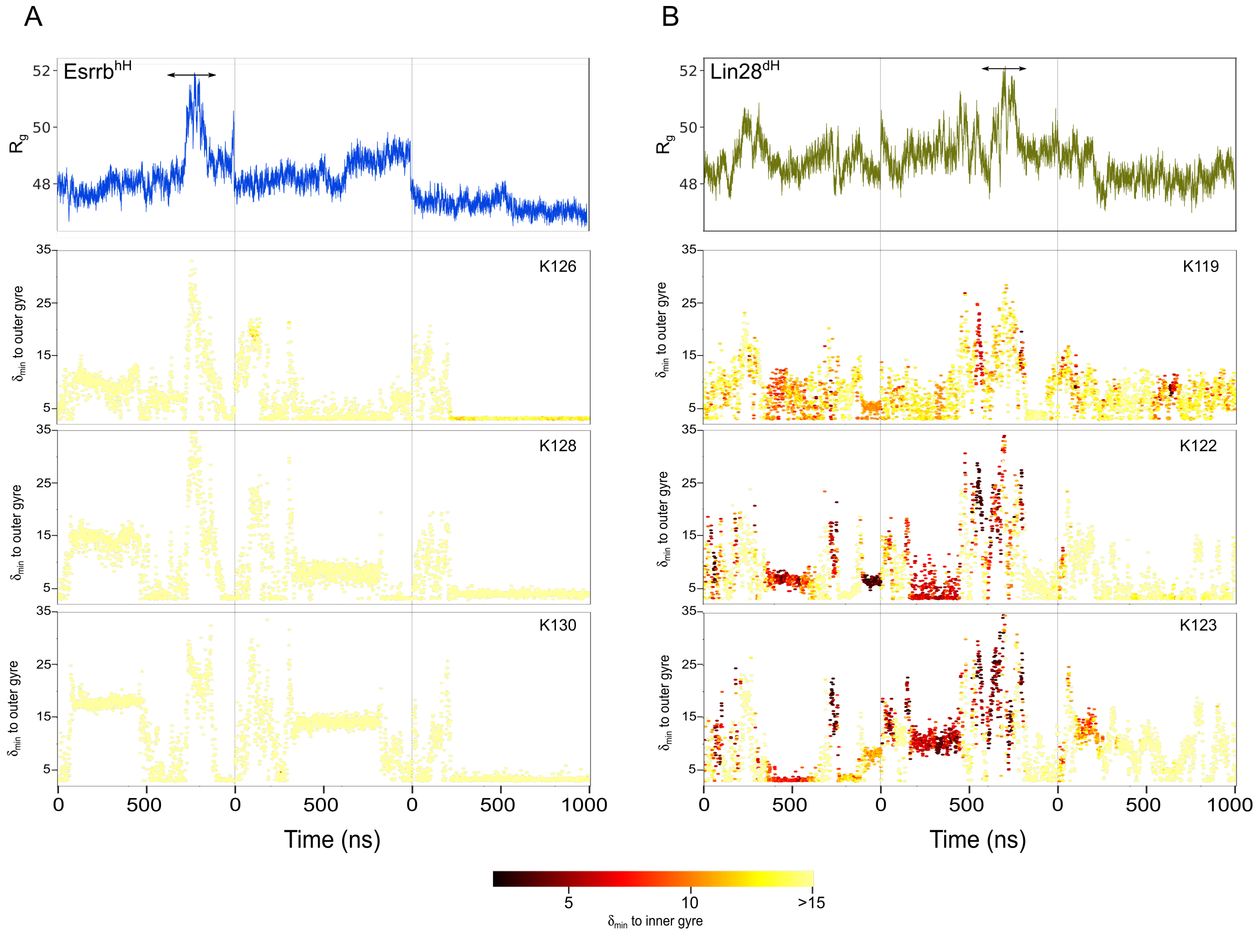

Supplement: S8 Fig — The evolution of the H2AC residues position relative to the inner and outer gyre. (A) The EsrrbhH nucleosome. (B) The Lin28bdH nucleosome. The plot at the top row shows the nucleosome radius of gyration to monitor opening and closing events. The other plots show the minimal distance of the residues to the outer gyre, colored by the minimal distance to the inner gyre of the DNA. (PDF) [file pcbi.1009013.s014.pdf]
