# Supplementary material for: Histone tails cooperate to control the breathing of genomic nucleosomes
Source: PLoS Comput Biol. 2021 Jun 3;17(6):e1009013. doi: 10.1371/journal.pcbi.1009013 (PMC8174689; doi:10.1371/journal.pcbi.1009013)
Supplement: Attachment — Submitted filename: Huertas_et_al_Response_to_Reviewers.pdf [file pcbi.1009013.s015.pdf]

Hubrecht Institute
Developmental Biology and Stem Cell Research

Vlad Cojocaru, Ph.D., Habil.
Hubrecht Fellow - Group Leader
In Silico Biomolecular Structure and Dynamics
Hubrecht Institute
Uppsalalaan 8, 3584 CT, Utrecht, The Netherlands
Tel: +31-30-212-1959;
Email: v.cojocaru@hubrecht.eu
https://www.hubrecht.eu/research-groups/cojocaru-group/

To: PLoS Computational Biology

Dear Prof. MacKerell,

We thank you and the Reviewers for evaluating our manuscript. We are submitting here a revised version in which we addressed all points and criticisms raised. We hope that our manuscript is now acceptable for publication. For the revisions we performed over 6 μs of additional simulations which provide further evidence for the mechanism we describe. Below we provide the detailed answers to each comment and we describe all the revisions we performed. The original comments are kept in italic blue for clarity. The modifications in the manuscript are in red.

Moreover, we have set a repository for providing access to all the simulations presented in this paper. For download the following password is required: “15v07l19a76d”

https://datashare.mpcdf.mpg.de/s/NpZBhKVCjxww32A

Yours sincerely,

Vlad Cojocaru (on behalf of all authors)

Reviewer #1: Authors investigated the roles of two histone tails in large scale opening motion (breathing) of nucleosomes using powerful atomistic molecular dynamics (MD) simulations. Three 1 microsecond long simulations of nucleosomes for two genomic DNAs with the two histone sets showed rare and large scale openings in a few events, while those for the 601 nucleosome did not show such a large breathing motion. Specific roles of the N-terminal tail of H3 and the C-terminal tail of H2A are carefully analyzed.

Strength: I consider it one of the most comprehensive atomistic MD works on nucleosomes to date, making it a very important contribution to the field. Personally, I learned a lot from the result and thus like this work very much. I think the conclusion make sense perfectly.

We thank the Reviewer for the appreciation of our work

Concern: However, due to its intrinsic hardness of the sampling, authors could observe only one event in Esrrb nucleosome and ~two(?) events in Lin28b nucleosome. I have a concern on the statistics and the robustness of the results, as will be explained below. Related to it, I would suggest one additional simulation to strengthen the paper.

Indeed, as the Reviewer points, the large opening events are rare in our simulations. We mainly describe the two largest openings we observed, one in each genomic nucleosome. The $Lin28b^{dH}$ nucleosome (Drosophila histones) opens at the 5' end whereas the $Esrrb^{hH}$ (Human histones) at the 3' end. In addition to these, we do observe smaller scale openings. Especially the $Lin28b^{dH}$ nucleosome opens reversibly in two of the three simulations. The $Esrrb^{hH}$ nucleosome also opens partially at the 5' end towards the end of simulation 2 (see the time series of the analyzed parameters in Figure S2). The mechanism of this opening is actually similar to the one we observed in $Lin28b^{dH}$ (most but not all contacts between the tails and the outer DNA gyre are lost but the H3 tail does not collapse on the DNA around the core DNA). We also observed a very short lived opening of the Widom nucleosome at the beginning of the simulation 2.

We agree that in the ideal situation, we'd have convergent trajectories revealing multiple, reversible transitions between largely open and tightly closed nucleosome conformations for all nucleosomes. This would ultimately enable the accurate quantification of the motions. However, convergence of nucleosome breathing in atomistic simulations would require very long simulation times, probably hundreds of microseconds to miliseconds. Such simulations are out of reach. On the other hand, the atomic resolution is needed to understand the interactions contributing to nucleosome breathing. Because of these considerations, we took the approach of performing multiple shorter simulations and combining them in ensembles. In this way, we aimed to simulate larger scale motions taking advantage of the different sampling of the conformational space in each individual simulation. The two large opening events as well as the smaller scale openings are all gradual and reversible, suggesting that they represent natural motions of these nucleosomes. This is also visible on the angle histograms in Figure 2, in which the large openings are revealed as a gradual expansions of the histograms in the direction of opening and not as isolated islands. We now highlighted the 2 largest opening events with bold arrows on these histograms.

To further strengthen our findings, we performed over 6 µs of additional simulations for the revised manuscript. In these, we could further reproduce the motions we observed in our original set of simulations. We describe the new results in new Figures 3 and in Supporting Figure S5 and explain them in the responses to the points below.

The specific comments:
1) Even though authors performed, in total, 18 microsecond MD runs, they found one large-scale opening event in Esrrb nucleosome and two? in Lin28b nucleosome. Based on the fact that this event is preceded by selected conformations of the tails, authors tried to conclude that "Transitions between open and closed nucleosome conformations were driven by the displacement and changes in compaction of the two histone tails."(from abstract, for example). However, looking at Fig. 6, for the Esrrb case, there is only one event only in 3' end linker DNA, at which the H3 tail was in the 6-th cluster. What does this mean? This can be just by chance, since this is the only one event. (I myself do not think it by chance, though)

We agree with the Reviewer that transitions between different H3 tail configurations are not occurring frequently in our simulations. However, this is what we expected in the first place. For example, the 45 residue long H3 tail in the crystal structure is in an extended conformation away from the DNA because the structure lacks linker DNA. Once we started simulating these nucleosomes with 11 base pairs of L-DNA at each end, the H3 tail collapsed on DNA in different configurations. Transitions between these configurations in 1 µs are rare. This is clearly seen in Figure 7 (former Figure 6). The less populated H3 clusters 4,5,6 (not only cluster 6) are specific for individual simulations. Nevertheless, we did observe a few large scale transitions, some of which were related to nucleosome breathing.

We consider that the only way to sample different histone tail configurations is to start multiple simulations and group them in ensembles. Of course, ideally we would run many independent simulations and not just three. And each simulation could be longer. However, because of the number of simulated systems, this would rapidly become unfeasible. Although it was a compromise to perform only three independent simulations per system, each only 1 µs long, we do provide one of the most extensive set of atomistic simulations of nucleosomes to date. This enabled us to observe large scale motions and analyze many different histone tail configurations.

For the particular case of the Esrrb^{hH} nucleosome, the collapse of the H3 tail on the core nucleosomal DNA, rather than the linker DNA allowed the large opening. As soon as the number of interactions between this tail, as well as the H2AC tail, and the outer DNA gyre increased, the nucleosome closed. We now show in additional simulations that a tail-less version of this nucleosome opens more extensively than the complete nucleosome but with a very similar pattern, the largest opening still occurring at the 3’ end. In addition, closed Esrrb^{hH} conformations opened in simulations started with H3

and H2AC configurations selected from the clusters that contain open nucleosome conformations. These additional simulations strengthen our findings that the large scale breathing motions of this nucleosome are mediated by H3 and H2AC tail dynamics. All new simulations and the modifications we implemented are described in a response below.

Unfortunately, we cannot compare it with the case of Lin28b because the clustering was done separately(which is unavoidable, though). Therefore, as it is, the appeal is simply way too strong.

As the Reviewer points, the clustering together of the Lin28b^{dH} and Esrrb^{hH} simulations is not possible. Nevertheless, the opening mechanism between the two nucleosomes is similar. In both cases it involves the loss of the interactions between the H3 and H2AC tails and the outer gyre of the DNA. However, we do agree that there are some important differences. In the case of Lin28b^{dH} a few of these contacts are kept and the H3 tail does not collapse on the core nucleosomal DNA around the dyad as in the case of Esrrb^{hH}. It is also worth noting that the Esrrb^{hH} nucleosome also undergoes a smaller scale opening at the end of simulation 2 with a mechanism that is more similar to the one observed in Lin28b^{dH}. This motion is incomplete because we stopped the simulation at 1 µs while the opening was taking place.

Related to the point, I would suggest an additional small MD runs. If the authors' view is correct, specific conformations of H3 and H2A C tails facilitate the large-scale opening. In a separate MD setup, they can restrain these tails near the conformations found in 700-800 ns of the first run of the Esrrb nucleosome, and conduct additional runs of 1 microseconds. If they find large-scale opening repeatedly, this markedly strengthens their appeal.

As already mentioned above, we appreciated the Reviewer's advice and we performed additional simulations to strengthen our paper and to provide further evidence for the mechanism we describe. These simulations are now presented in the new Figure 3 and the new Supporting Figure S5. In designing these simulations, we reasoned that restraining the positions of the tails to keep them in or near the configurations found in the open nucleosome conformations would force the entire nucleosome to adapt its dynamics to the fixed tail positions. Therefore, the dynamics would be altered in an artificial way. To overcome this limitation, we took two alternative approaches:

I. We performed new simulations with the three tail-less nucleosomes, 1 µs long each. These simulations are now presented in the new main Figure 3. They revealed that the tail-less nucleosomes open faster, for longer times and more extensively. Previous studies have shown that tail-less nucleosomes breath more extensively, but these studies were performed at high salt concentrations, or at high temperature and with engineered DNA sequences. Remarkably, in our simulations the Lin28-TL and Esrrb-TL nucleosomes breath extensively in standard conditions: 150 mM salt, 300 K. Moreover, the breathing occurs with a similar pattern as observed in complete nucleosomes. Lin28b-TL opens

more at the 5’ end whereas the Esrrb-TL displays enhanced opening at the 3’ end. This suggests not only that histone tails are crucial for keeping the nucleosome closed but also that the opening pattern of the nucleosome is encoded in the DNA sequence. As the H3 and H2AC tails are the only tails located near the L-DNAs, the simulations of tail-less nucleosomes strengthen our findings regarding the role of these two tails. We modified the text at different places to include the results from the tail-less simulations. In the Abstract we introduced a sentence, and the results are summarized at the end of Introduction, in lines 90-95. In the Results we modified two paragraphs at page 7 (lines 229-237) to present these new findings which we also discuss in the Discussion at page 13, lines 465-467 and at page 14, lines 535-538.

II. We performed new simulations of both Lin28b^{dH} and Esrrb^{hH} started with a closed nucleosome but with the H3 and H2AC tails selected from the clusters that contain open nucleosomes. First, we performed unrestrained simulations with these modified nucleosomes (500 ns each) because we considered this to be the least biased approach. Remarkably, the Lin28b^{dH} nucleosome opened extensively along the direction defined by both γ angles in 2 out of the 3 simulations (see new Supporting Figure S5), and only along one of these directions in the third simulation. On the other hand, the Esrrb^{hH} nucleosome remained closed in these initial simulations because the H3 tail reformed its interactions with the outer DNA gyre rapidly during the equilibration. The reason for this was that the H3 tail configuration established in the open nucleosome was very near the outer DNA gyre of the closed nucleosome we selected. Therefore, we took an alternative approach in which we first used Modeller to remodel a part of the H3 tail to ensure it is further away from the outer gyre. The remodeling moved slightly the tail without changing its configuration giving more room for DNA motions. Then, we performed an initial 8 ns long biased MD simulation in which we forced the minimal interatomic distance and the number of contacts between the H3 and H2AC tails and the outer DNA gyre to adopt values that are incompatible with direct interactions that would keep the nucleosome closed. In this biased simulation, the very closed nucleosome (Rg ~ 46) opened only marginally and only along γ_2 (see new Supporting Figure S5). After this initial biased simulation, we started again unrestrained MD simulations. Remarkably, in one of these simulations the Esrrb^{hH} nucleosome opened as in our original simulation and the open conformation stabilized in the second half of the simulation (see new Supporting Figure S5). In a second simulation Esrrb^{hH} also opened but less than in the first simulation and slightly less than in the original simulation. In a third simulation Esrrb^{hH} remained closed due to the reformation of the interactions between the tails and the outer DNA gyre. In two extra simulations which are not included and are still running (did not reach 500 ns), we observed yet again extensive opening of Esrrb^{hH}. These results demonstrate that we are able to reproduce the opening of Esrrb^{hH} when the H3 and H2AC tails are in configurations compatible with nucleosome opening. The fact that it has been more difficult to reproduce the opening of the Esrrb nucleosome is not surprising. In our previous study (Huertas et al 2020, Biophys. J.) we showed in experiments that the Esrrb nucleosome disassembles at temperatures intermediate between the Lin28b and the Widom nucleosomes. We describe these simulations and the findings from them in the Supporting Information (see Supporting Methods, Supporting Data, and Supporting Figure S5). We

also refer to them in the main text at page 9, lines 312-315. We decided to not include the details of these simulations in the main article because although they do provide excellent support for our findings, they were performed with artificially selected initial structures which biased the dynamics of the nucleosomes.

In another set of simulations that we do not show in this manuscript which we performed to investigate the effect of the binding of the transcription factor Oct4 on the dynamics of Esrrb and Lin28b, we also observed that Oct4 induced nucleosome breathing requires similar configurations of the H3 and H2AC tails as the ones we report here. These simulations are presented in a follow up manuscript, now in peer review.

Taken together, all these data provide solid evidence for the histone tail mediated nucleosome breathing mechanism we present in this manuscript.

2) I thought the current way of presenting the result unnecessarily complicated. This may be partly because of the numbering in the cluster analysis. Currently, authors sorted 8 clusters, probably, by their sizes, which is automatic, but does not help our understanding much. I would suggest the ordering by some physical parameter. For example, the DNA clusters can be sorted by the gamma_1 gamma_2 in 5' end and gamma_2-gamma_1 in 3' end, which is much more intuitive. The other two clusters for the tails can be sorted based on the data in Fig. 7 (Differences in the distances of R2-K14 between inner and outer gyres, for example).

We thank the Reviewer for this suggestion. However, we consider that the ranking of the clusters by their size is the most comprehensive way to present these data. In case we rank the clusters using a physical parameter such as the R_g or the γ angles, we will lose the objective classification by the number of snapshots that are present in each cluster. This classification shows clearly that the clusters with open nucleosome conformations are less populated than the clusters with closed nucleosome conformations. The histograms with the clusters labeled in Figure 5 (former 4) helps the reader to assign a cluster number to specific nucleosome conformations.

3) I really eager to see one nice scattered plot that directly indicated the correlation between the tail conformations and the degree of opening. For the latter, one can use gamma_1 - gamma_2 as a simple score. Or, RoG itself is a nice score. For the tail conformations, I think, again, that the data in Fig. 7 can be rearranged to make one score (an order parameter) that monitors if the tail is directed to the outer gyre/linker DNA or to the dyad region. With the two scores in x- and y-axis, one can make the scattered plot showing their correlation, I think.

We now include scattered 3D dot plots of the Rg with respect to the number of contacts between the H3 and H2AC tails and the outer DNA gyre. These plots nicely show that the tail – DNA contacts are lost before the nucleosome opens. These plots are included in the new main Figure 7 (former 6), and described in the text in lines 384-387 at page 11. In addition, in the Supporting Figure S6 (former S5) the principal component analysis also shows the correlation between nucleosome breathing and histone tail dynamics.

4) I find it surprising that the nucleosome dynamics is rather different between hH and dH. Can authors argue why so? What part of difference caused this difference?

We agree with the Reviewer's point that there are some significant differences between the simulations with the Drosophila and the Human histones. However, we would like to emphasize again that the opening mechanism observed in the Lin28b^{dH} and Esrrb^{hH} nucleosome are rather similar. In both cases, the opening occurred when the H3 and H2AC tails established a few or no interactions with the outer DNA gyre. The clustering showed also remarkably similar sampling between the dH and hH simulations. The cluster distributions for the 3' end of the Esrrb^{dH} and the 5' end of the Lin28^{dH} where the openings occur are almost symmetric (see Figure 5, former Figure 4).

Our data suggests that the differences observed between the dH and hH simulations of each nucleosome are mainly due to the sampling of the histone tail configurations in different simulations and not so much influenced by the differences between the Drosophila and the Human histones. In most cases, the differences between the 3 individual simulations of each nucleosome are at least as large as the differences between hH and dH simulations. Perhaps with the exception of the Windom nucleosome which is remarkably stable in all simulations. Therefore, we argue that most of the differences we observed are due to the lack of convergence of histone tail dynamics which is notoriously hard to achieve.

Having said that, there is a small difference in the length and charge of the H2AC tail between the Drosophila and Human histones. We do see that this difference is relevant for the behavior of this tail (see Fig. 7, former Fig 6). This difference may explain the fact that the Lin28b^{dH} nucleosome opens even when some contacts of the H3 tail with the outer DNA gyre are kept. Also, the fact the the human H2AC is longer may also explain why the opening of the Esrrb^{hH} nucleosome was harder to reproduce than that of the Lin28b^{dH} nucleosome. In some of new simulations we report in the new Supporting Figure S5, we observed that the longer human H2AC tail reached out to interact with the outer DNA gyre when the Esrrb^{hH} nucleosome was in process of opening. In some cases, these interactions prevented the nucleosome from opening further.

Reviewer #2: Chromatin remodeling is cucial for proper expression of the genetic information in eukaryotes, and opening of the nucleosome is an important step in this process, which is however\ difficult to study experimentally. Here extensive all-atom molecular dynamics simulations are used to shed light on the role of interactions between histone tails and nucleosomal DNA in the opening of the nucleosome. The main result of the study is that there does indeed seem to be a relation between tail conformations of two of the histones and conformations of the DNA when there is an opening motion in the nucleosome.

As the authors note, the simulations are however too short (3 x 1 microsecond for each system) to be converged wrt to nucleosome opening; opening events of short (100 ns) duration are only observed in two out of a total of 18 simulations. It is therefore not really possible to draw any conclusions regarding differences between the DNA sequences used in terms of their ability to support opening events.

We do agree with the Reviewer that our simulations are too short and they do not reach convergence. We specified this very clearly in our manuscript and also in response to Reivewer 1. However, we disagree with the Reviewer that we cannot draw any conclusions from these simulations. The mechanism of both large opening events observed in the two different genomic nucleosomes are similar as explained above. Both extensive openings require specific positions and conformations of the H3 and H2AC tails that form few or no interactions with the outer DNA gyre. Such large openings do not occur in the Widom nucleosome. We also showed in our previous study (Huertas et al. 2020, Biophys J) that in thermic disassembly experiments Lin28b disassembles at the lowest temperature and Widom at the highest. These experiments also show that the DNA sequence plays a significant role in addition to the role of the H3 and H2AC tails we describe in this manuscript.

Moreover, in the revisions we also performed simulations with the three tail-less nucleosomes (see detail in the response to Reviewer 1). The opening of these nucleosomes was faster, longer lived and more extensive than that of the complete nucleosomes. However, the pattern of the opening was very similar with that we observed in the complete nucleosomes. Lin28b-TL opened more at the 5' end and Esrrb-TL opened more at the 3' end. This shows that the opening is actually encoded in the DNA sequence and that the two tails function to keep nucleosomes closed. These data is now presented in the new Figure 3.

In addition, as described in detail in the response to Reviewer 1, we were also able to reproduce the openings of both genomic nucleosomes in simulations started with a closed nucleosome conformation but with H3 and H2AC tail configurations selected from the clusters containing open nucleosome conformations.

Therefore, despite insufficient sampling and lack of convergence which did not allow us to sample multiple, reversible transitions between very open and very closed conformations of complete nucleosomes, all our data taken together provide sufficient evidence for the mechanism of histone tail mediated nucleosome breathing we describe.

Three different DNA sequences have been used, two "genomic" and one "engineered". The rationale for choosing these sequences is not given in the manuscript, nor is the difference of the "engineered" sequence from the other two described; in particular it would be of interest to know if this DNA differs in its ability to interact with the relevant histone tails.

We did describe the rational behind the choice of the nucleosome sequences in the Manuscript in in the before the last paragraph of the Introduction. Now, we further clarified this at page 3, lines 79-90. The Widom sequence was chosen since it's the artificial sequence used in the vast majority of nucleosome studies, due to it's high affinity and easier manipulation in vitro. However, it's an engineered sequence that doesn't reflect the real behavior of nucleosomes in the genome. Also crystal structures of the Widom nucleosomes without tails are available. On the other hand, the native sequences were chosen from the studies by Soufi et al (2012 and 2015) in which nucleosome positioning was overlapped with the genomic binding profile of the transcription factor Oct4, a master regulator of stem cell pluripotency, crucial for the conversion of somatic cells to pluripotent stem cells. In these studies, the Lin28b and Esrrb nucleosomes were shown to be bound by Oct4. We used the genomic data from Soufi et al and biochemical data by our collaborators to build models of the two genomic nucleosomes which we validated by monitoring the accessibility of the known Oct4 binding sites. We then used these nucleosomes in the simulations we present in this manuscript.

The study we present here is part of our more general effort to understand how Oct4 interacts with genomic nucleosomes and what role nucleosome dynamics play in this interactions. Our initial study addressing this question was published in the Biophysical Journal in May 2020 and a second manuscript about the topic is now in peer review. When analyzing all the simulations of the free nucleosomes we performed to compare with the Oct4 bound nucleosomes, we observed the histone tail mediated nucleosome breathing mechanism we describe in this manuscript.

The results in figure 2 do not convincingly show any significant/important differences in the behavior between the different systems. Is the area of the distributions really an important observable, in particular since the peripheral regions of the distributions are not very highly populated?

We disagree with the Reviewer that Figure 2 does not reveal the differences we analyzed. We clearly labeled the histograms to point the opening-closing direction in the γ_1/ γ_2 space by arrows in the inserts.

The two large opening events clearly expand the histogram areas in the case of the Lin28b^{dH} and Esrrb^{hH} nucleosomes. For further clarifying this, we now added 2 additional bold arrows that point to the large openings in the two corresponding histograms.

The values for the histogram areas are used as an indicative measure of the degree of sampling in different simulations. The extremities of these histograms represent the rare opening events and in most cases these extremities are continuous extensions of the histograms and not isolated islands. Therefore, we consider that the areas enclosing the complete data are relevant for the purpose for which they are used.

The discussion of epigenetic histone modifications is relevant, and it would strengthen the manuscript to have simulation data for nucleosomes with either or both of the histones of interest modified.

We agree with the Reviewer that simulations with modified histone tails is the perfect follow-up for this study. However, here we do not focus on how modifications affect nucleosome dynamics but on the role of the unmodified histone tails.

Is the radius of gyration wrt to an axis or wrt the center of mass? Please also note that it is usually denoted Rg, where the g is a subscript - introducing a new symbol "RoG" is just confusing.

The radius of gyration was calculated in reference to the center of mass. This is now clearly indicated in the schematic representation in Figure 2. We also changed "RoG" to "Rg" throughout the manuscript

Since three replica have been simulated for each system it should be possible to provide error estimates for quantities of interest.

We provide the complete time series for all the relevant parameters we analyzed in all simulations (see Figures 4, 7, Supporting Figures S1, S2, S3, S7, S8, and Supporting Document S3 in the revised manuscript). Because the large opening events we observed are rare events, the distributions of the calculated parameters are not normal distributions. Therefore, measures such as standard deviation and standard error of mean do not provide an accurate representation of the data. The percentiles 5 to 95, together with the complete time series, provide a more complete representation of the data.

Have all data underlying the figures and results presented in the manuscript been provided?
Large-scale datasets should be made available via a public repository as described in the PLOS

Computational Biology data availability policy*, and numerical data that underlies graphs or summary statistics should be provided in spreadsheet form as supporting information.*

Reviewer #1:Yes

Reviewer #2:None

We have set a repository for providing access to all the simulations presented in this paper. For download before publication the following password is required: “15v07l19a76d”. After publication, the download will be password free.

https://datashare.mpcdf.mpg.de/s/NpZBhKVCjxww32A
